# Analysis of Various Subsets of Circulating Mononuclear Cells in Asymptomatic Coronary Artery Disease

**DOI:** 10.3390/jcm2030032

**Published:** 2013-07-30

**Authors:** Alexander E. Berezin, Alexander A. Kremzer

**Affiliations:** 1 Internal Medicine Department, State Medical University, Zaporozhye UA-69121, Ukraine; 2 Clinical Pharmacology Department, State Medical University, Zaporozhye UA-69121, Ukraine; E-Mail: kremzer@gmail.com

**Keywords:** circulating mononuclear cells, asymptomatic coronary artery disease, cardiovascular risk factors

## Abstract

The objective of this study was to evaluate the correlation between multiple cardiovascular risk factors (MCRFs) and circulating mononuclear cells (CMCs) in asymptomatic coronary artery disease patients. Design and Methods: 126 subjects (54 male), aged 48 to 62 years, with asymptomatic coronary artery disease (CAD) documented previously with angiography, and 25 healthy volunteers were enrolled in the study. The flow cytometric technique was used for predictably distinguishing cell subsets that depend on the expression of CD14, CD34, Tie-2, CD45, and CD309 (VEGFR2). Results: The analysis of the outcome obtained shows a trend of an increase in circulating CD45^−^CD34^+^ CMCs and a reduction in CMC population defined as CD14^+^CD309^+^ and CD14^+^CD309^+^Tie^2+^ in known asymptomatic CAD patients in comparison with healthy volunteers. Substantial correlations between CD45^−^CD34^+^ and conventional cardiovascular risk factors (hs-CRP, T2DM, serum uric acid and hypertension) were found in the patient cohort. The concentrations of CD14^+^CD309^+^ and CD14^+^CD309^+^Tie^2+^ CMCs had effect on such factors as T2DM (RR = 1.21; 95% CI = 1.10–1.40; *p* = 0.008), hs-CRP > 2.54 mg/L (RR = 1.29; 95% CI = 1.12–1.58; *p* = 0.006), Agatston score index (RR = 1.20; 95% CI = 1.15–1.27; *p* = 0.034), and occurrence of three and more cardiovascular risk factors (RR = 1.31; 95% CI = 1.12–1.49; *p* = 0.008). Conclusion: It is postulated that the reduction in circulating CD14^+^CD309^+^ and CD14^+^CD309^+^Tei^2+^ CMCs is related to a number of cardiovascular risk factors in asymptomatic patients with known CAD.

## 1. Introduction

Multiple cardiovascular risk factors (MCRFs) in cardiovascular diseases, such as chronically increased blood pressure, hyperlipidemia, hyperuricemia, hyperglycemia, and obesity, have a negative impact on the heart exposed to ischemia [1,2]. It has been postulated that hematopoietic-derived progenitor cells have an effect on angiogenesis and tissue repair following several injuries, such as ischemia, reperfusion, inflammation, allograft vasculopathy, atherosclerosis, stroke, *etc*. [3,4,5,6]. Many studies have demonstrated the presence of circulating endothelial progenitor cells (EPCs) in the peripheral circulation [5,7]. Circulating EPCs have been defined previously using two distinct methods to characterize them, *i.e.*, flow cytometry and cell culture; recently, however, the validity of both definitions has been questioned. Flow cytometric definition is based on the expression of CD34^+^ on the circulating cells. Usually, the recent studies using flow cytometry have investigated cells that express CD34^+^ and CD309^+^(VEGFR-2^+^). An alternative definition of EPCs describes colonies of spindle-shaped cells appearing after culture of blood-derived mononuclear cells. Сirculating EPCs, however, might not only have bone-marrow origin; they are also transformed from peripheral mononuclear cells that have the capacity to come to the sites of tissue injury and may be differentiated into mature endothelial cells [8,9]. It has been determined that EPCs have been largely characterized by the expression of the primitive hematopoietic progenitor markers CD34^+^ and VEGF receptor (VEGFR)-2 [10,11]. Basically, CD34^+^CD45^−^ cells have nonhematopoietic origin; and recently they have been identified as putative EPCs by virtue of their ability to form “late outgrowth” colonies phenotypically and functionally indistinguishable from mature endothelial cell colonies in culture [12,13]. Previous studies have shown an increase in the circulating concentration of population CD34^+^CD45^−^ cells in patients with established coronary artery disease (CAD) and peripheral artery disease [10,14,15]. Theoretically, CD34^+^ cells which do not express CD45 by flow cytometry may include cells capable of developing *bona fide* endothelial cells. Nevertheless, traditional EPC populations, such as CD34^+^VEGFR-2^+^ and CD34^+^VEGFR-2^+^CD133^+^, are not related to severity of CAD or clinical outcome in the patients with acute coronary syndrome and unstable angina. On the other hand, the concentrations of proangiogenic monocites may reflect the extent of vascular injury and atheroma burden in this patient population [3,9]. The objective of this study was to evaluate the correlation between MCRF and various types of circulating mononuclear cells (CMCs) that express CD14^+^VEGFR-2^+^ and CD14^+^VEGFR-2^+^Tie^2+^ in patients with asymptomatic coronary artery disease.

## 2. Design and Methods

### 2.1. Study Population

The study population was structured retrospectively after determining the coronary artery disease (CAD) by contrast-enhanced spiral computed tomography angiography in 126 asymptomatic subjects. Twenty five healthy volunteers were enrolled in the study aimed at the verification of the reference average of biological markers. All the subjects gave their written informed consent to participate in the study prior to enrollment. The following are the exclusion criteria: Symptomatic chronic heart failure; left ventricular ejection fraction (LVEF) ≤40%; uncontrolled diabetes mellitus; severe kidney and liver diseases that may affect clinical outcomes; malignancy; unstable angina; Q-wave and non-Q-wave MI within 30 days before the study entry; creatinin plasma level above 440 μmol/L; eGFR index <35 mL/min/m^2^; brain injury within three months before the enrollment; body mass index above 30 kg/m^2^ and less than 15 kg/m^2^; pulmonary edema; tachyarrhythmia; valvular heart disease; thyrotoxicosis; ischemic stroke; intracranial hemorrhage; acute infections; surgery; trauma; all ischemic events within the three previous months; inflammations within the previous month; neoplasm; pregnancy; implanted pacemaker, any disorder that may discontinue the patient’s participation in the study according to investigators; and finally the patient’s refusal to participate in the study or to give his consent for it. 

### 2.2. Contrast-Enhanced Spiral Computed Tomography Angiography

The coronary vessel-wall, plaque geometry, and compositional parameters were measured on contrast-enhanced spiral computed tomography (CT) angiography [16]. Contrast-enhanced spiral CT was performed on a Somatom Volum Zoom scanner (Siemens, Erlangen, Germany) with two rows of detectors (32 × 2 CT system) at the time of end-expiratory breath-hold. After noncontrast localization image acquisition, injection of Omnipak nonionic contrast (Amersham Health, Carrigtohill, Ireland) was used to determine the optimal coronary arterial image. Images were reconstructed in 0.6-mm axial slices. The coronary artery calcification was quantified by calculating the Agatston score index and measuring the calcification mass [17]. Calcified atherosclerotic plaque (CAP), high-density noncalcified plaque (HD-NCP), and low-density noncalcified plaque (LD-NCP) were determined. Calcified atherosclerotic plaques were characterized by an attenuation value that was 150 HU (Hounsfield units) or greater for CAP, 30 to 149 HU for HD-NCP U, and 100 to +30 HU for LD-NCP [18,19]. 

### 2.3. Echocardiography Examination

According to recommendation of the American Society of Echocardiography, standard transthoracic echocardiography in B-mode was performed on an ACUSON scanner (Siemens, Erlangen, Germany) using a transducer with a frequency of 2.5–5 MHz. End-diastolic and end-systolic LV volumes were obtained using a two-dimensional reference sector according to Simpson’s method, and the LV ejection fraction (LVEF) was calculated according to conventional methods [20].

### 2.4. Glomerular Filtration Ratio Estimation

Estimated glomerular filtration ratio (eGFR) was calculated using the MDRD formula [21].

### 2.5. Blood Sampling and Biomarker Measurements

All the samples were placed into EDTA tubes with serum gel for further flow cytometry analysis and mononuclear cell preparation. The whole blood was analyzed: The complete blood count with white blood cell differential count was carried out using the analyzer. After taking for blood chemistry test, all the blood samples were placed in the cooling vacutainer and centrifugated immediately (at a temperature of 4 °C at 6000 rpm for 15 min). After centrifugation, the serum was coded and stored in the refrigerator at a temperature of −70 °C until used.

#### 2.5.1. High-Sensitive C-Reactive Protein Level Determination

High-sensitive C-RP (hs-C-RP) level was measured by a nephelometric technique and obtained with a “AU640 Analyzer” (Olympus Diagnostic Systems Group, Shizuoka-ken, Japan).

#### 2.5.2. Serum Uric Acid Measurement

Serum uric acid level (SUA) was determined by enzymatic methods using a Beckman Synchron LX20 chemistry analyzer. The analytical average range for SUA was 0.5–12 mg/dL.

#### 2.5.3. Cholesterol Level Measurement

Concentrations of total cholesterol (TC) and high density lipoprotein (HDL) cholesterol were determined with the Dimension Clinical Chemistry System (Dade Behring Inc., Newark, NJ, USA). Low density lipoprotein (LDL) cholesterol was calculated using the Friedewald formula [22].

#### 2.5.4. Circulating EPCs

The flow cytometric technique (FCT) was used for predictably distinguishing circulating cell subsets, which depend on expression of CD45, CD34, CD14, Tie-2, and VEGFR2, using High-Definition Fluorescence Activated Cell Sorter (HD-FACS) methodology [23]. Accordingly, the cells in question were phenotyped on the basis of their forward scatter characteristic (FSC) and side scatter characteristic (SSC) profiles. The cells were directly stained and analyzed for phenotypic expression of surface proteins using anti-human monoclonal antibodies, including anti-CD45 FITS (BD Biosciences, San Jose, CA, USA), anti-CD34 FITS (BD Biosciences), anti-VEGFR-2 known as anti-CD309 (BD Biosciences), anti-Tie2 (BD Biosciences) and anti-CD14 (BD Biosciences). The fluorescence minus one technique was used to provide negative controls and establish positive stain boundaries. After lysis of erythrocytes with Utilize wash solution, the samples were centrifuged at 200× *g* for 15 min; then they were washed twice with PBS and fixed immediately. 

Double- or triple-positive events were determined using Boolean principles (“and”, “not”, “or”, *etc*.). Circulating EPCs were defined as CD34/VEGFR2 positive cells with lack of CD45 expression. From each tube 500,000 events were analyzed. For CD14^+^ populations, coexpression with Tie-2^−^ and/or VEGFR-2^−^ was determined using quadrant analysis. Mononuclear cells were cultured for functional analysis (CFUs) after FCT. Standardized cell counts were presented as a percentage of the total of the white blood cells count, identified as the total number of all CD45^+^ cells.

#### 2.5.5. Cardiovascular Risk Calculation

A 10-year cardiovascular risk for study patients was calculated using the Framingham General Cardiovascular Risk Score (2008) by on-line calculator.

## 3. Statistical Analysis

All the statistical analyses were performed in SPSS for Windows v. 20.0 (SPSS Inc., Chicago, IL, USA, 2011). Continuous variables are presented as mean ± SD, mean and 95% CI or median and interquartile range. Categorical variables are expressed as frequencies and percentage. An independent group *t*-test was used to compare all the interval parameters matching the criteria of normality and homogeneity of variance. For interval parameters that fail to match these criteria, the non-paramentric Mann-Whitney test was used to compare variables. Categorical variables and frequencies were compared using Chi^2^ test and Fisher exact test of independence. SUA frequencies were normally distributed (using the Kolmogorov-Smirnov test), and data were not positively skewed. Frequencies of CMCs and hs-CRP concentrations were not distributed. The data, however, were not transformed. The potential factors that may be associated with CMCs were identified first with the univariate analysis (ANOVA), and then the independent predictors of a decrease in CMCs were searched with the multivariate one-step backward logistic regression analysis, initially including variables for which a *p* value < 0.1 was achieved from the univariate analysis. The odds ratio (RR) and confidence intervals (95% CI) were calculated for factors independently associated with a decrease in circulating CMCs. A calculated difference of *p* < 0.05 was considered significant. 

## 4. Results and Discussion

General characteristics of study patients are presented in Table 1. Two groups of persons surveyed (healthy volunteers and patients with known CAD) were compared in terms of demographics, smoking, body mass index, CAD family history, mean systolic BP, heart rate, creatinin, fasting glucose and eGFR. Patients with known CAD, however, had hyperlipidaemia (44.4%), arterial hypertension (66.7%), T2DM (36.5%). An increase in HbA1c, serum uric acid, hs-CRP, LDL cholesterol, and triglycerides, as well as a decrease in HDL cholesterol were found in patients with known CAD when compared with healthy volunteers. Mediana of a 10-year Framingham General Cardiovascular Risk in patients with known CAD and in healthy volunteers was 23% and 2% respectively.

Baseline angiographic and treatment characteristics of patients with known CAD are presented in Table 2. Calcified atherosclerotic plaques were determined in 96% of patients; HD-NCP and LD-NCP were found in 31% and 25% respectively. The median of the Agatston score index was 586 (95% CI = 401–838). Coronary arteries with plaques were determined in 36.5%; 33.3%; and 20.2% for one vessel, two vessels, three and more vessels respectively. 

**Table 1:** General characteristics of study patients.

	Healthy volunteers (*n* = 25)	Patients with known CAD (*n* = 126)	*p* Value
Age, years	51.70 ± 6.10	58.34 ± 9.60	0.22
Male, *n* (%)	14 (56.0%)	74 (58.7%)	0.56
Framingham General Cardiovascular Risk, %	23 (16–27)	2 (1–3)	0.001
Arterial hypertension, *n* (%)	None	84 (66.7%)	-
Hyperlipidaemia, *n* (%)	None	56 (44.4%)	-
T2DM, *n* (%)	None	46 (36.5%)	-
Premature CAD, *n* (%)	2 (8.0%)	12 (9.5%)	0.48
Smoking, *n* (%)	6 (24.0%)	26 (20.6%)	0.42
Body mass index, kg/m^2^	23.3 (95% CI = 20.1–25.1)	24.1 (95% CI = 21.6–28.7)	0.58
eGFR, mL/min/m^2^	93.5 (95% CI = 88.3–101.3)	82.3 (95% CI = 68.7–102.6)	0.21
HbA1c, %	3.8 (95% CI = 3.1–4.6)	6.8 (95% CI = 4.1–9.5)	0.001
Fasting glucose, mmol/L	4.11 (95% CI = 3.2–5.5)	5.20 (95% CI = 3.3–9.7)	0.07
Creatinin, μmol/L	65.7 (95% CI = 53.1–80.5)	72.3 (95% CI = 58.7–92.6)	0.48
SUA, mmol/L	17.1 (95% CI = 9.1–25.7)	23.8 (95% CI = 15.8–31.3)	0.05
hs-CRP, mg/L	1.15 (95% CI = 0.11–3.18)	4.95 (95% CI = 3.15–9.80)	0.001
TC, mmol/L	4.1 (95% CI = 3.1–5.0)	5.1 (95% CI = 3.9–6.1)	0.012
LDL cholesterol, mmol/L	2.75 (95% CI = 2.44–3.6)	3.23 (95% CI = 3.11–4.4)	0.014
HDL cholesterol, mmol/L	1.01 (95% CI = 0.92–1.2)	0.91 (95% CI = 0.89–1.12)	0.012
Mean systolic BP, mm Hg	127.30 ± 5.66	130.90 ± 8.41	0.44
Heart rate, beats per min	68.56 ± 3.17	70.52 ± 3.34	0.52
LV EF, %	65.40 ± 0.87	42.80 ± 0.76	0.001

Note: CAD—coronary artery disease, CI—confidence interval, T2DM—type 2 diabetes mellitus, eGFR—estimated glomerulal filtration ratio, TC—total cholesterol, HbA1c—glycated hemoglobin, LDL—low-density cholesterol, HDL—high-density cholesterol, SUA—serum uric acid, BP—blood pressure, hs-CRP—high sensitive C-reactive protein, LV EF—left ventricular ejection fraction.

All the asymptomatic patients were informed about coronary angiography, and they were treated according to current clinical guidelines with diet, lifestyle modification, and drug therapy that included ACE inhibitors/ARBs, aspirin or other antiagregants, statins and metformin if needed.

Analysis of the outcomes obtained showed no significant increase in circulating CD34+ subset cells (CD45^+^CD34^+^ and CD45^−^CD34^+^) in known asymptomatic CAD patients when compared with healthy volunteers (Table 3). The authors suppose that there are decreased CD14^+^ subsets of CMCs in CAD subjects. Indeed, there is a significant difference between the medians of CD14^+^CD309^+^ and CD14^+^CD309^+^Tie^2+^ in both healthy volunteers and patients with known asymptomatic CAD respectively.

There were no significant correlations between CD45^+^CD34^+^ and demographics, cardiovascular risk factors, Agatston score index, and plaque characteristics in a univariate regression model. The relationship between CD45^−^CD34^+^ cells subset frequency and high sensitive C-reactive protein (*R* = 0.864; *p* = 0.001), T2DM (*R* = 0.614; *p* = 0.001), Agatston score index (*R* = 0.467; *p* = 0.001), SUA (*R* = 0.380; *p* = 0.002), and arterial hypertension (*R* = 0.240; *p* = 0.026) was determined by positive linear regression. A negative correlation was supposed between CD45^−^CD34^+^ cell subset frequency and LD-CAP (*R* = −0.508; *p* = 0.001), LVEF (*R* = −0.414; *p* = 0.001) and smoking (*R* = −0.222; *p* = 0.040). There is a significant positive correlation between CD14^+^CD309^+^ cell subset frequency and high sensitive C-reactive protein (*R* = 0.892; *p* = 0.001), Agatston score index (*R* = 0.520; *p* = 0.001), T2DM (*R* = 0.520; *p* = 0.001), SUA (*R* = 0.348; *p* = 0.002), LDL-cholesterol (*R* = 0.322; *p* = 0.001), arterial hypertension (*R* = 0.280; *p* = 0.006) and TC (*R* = 0.260; *p* = 0.041). A negative correlation between CD14^+^CD309^+^ cell subset frequency and LD-CAP (*R* = −0.591; *p* = 0.001), LVEF (*R* = −0.424; *p* = 0.001), smoking (*R* = −0.259; *p* = 0.042), and CD14^+^CD309^+^Tie^2+^ cell subset frequency was found, as well as correlation with high sensitive C-reactive protein (*R* = 0.92; *p* = 0.001), Agatston score index (*R* = 0.538; *p* = 0.001), T2DM (*R* = 0.597; *p* = 0.001), SUA (*R* = 0.382; *p* = 0.002), LDL-cholesterol (*R* = 0.354; *p* = 0.001), TC (*R* = 0.258; *p* = 0.043), CAP (*R* = −0.598; *p* = 0.001), LD-CAP (*R* = −0.594; *p* = 0.001), LVEF (*R* = −0.374; *p* = 0.001), smoking (*R* = −0.285; *p* = 0.042) and BMI (*R* = −0.272; *p* = 0.046).

**Table 2:** Baseline Angiographic and Treatment Characteristics of patients with known CAD.

	Patients with known CAD (*n* = 126)
CAP, *n* (%)	96 (95% CI = 31–102)
HD-NCP, *n* (%)	31 (95% CI = 21–56)
LD-NCP, *n* (%)	25 (95% CI = 13–48)
Agatston score index	586 (95% CI = 401–838)
Coronary arteries with plaques determined	
1 vessel, *n* (%)	46 (36.5%)
2 vessels, *n* (%)	42 (33.3%)
3 vessels and more, *n* (%)	38 (30.2%)
ACEI/ARBs, *n* (%)	126 (100%)
Aspirin, *n* (%)	98 (77.8%)
Other antiagregants, *n* (%)	6 (4.8%)
Statins, *n* (%)	94 (74.6%)
Metformin, *n* (%)	41 (32.5%)

Note: CI—confidence interval, ACEI—angiotensin-converting enzyme inhibitor, ARBs—angiotensin-2 receptor blockers, HD-NCP—high-density noncalcified atherosclerotic plaque, LD-NCP—low-density noncalcified atherosclerotic plaque, CAP—calcified atherosclerotic plaques.

The potential factors, which may be associated with EPCs determined as CD45^−^CD34^+^, CD14^+^CD309^+^, and CD14^+^CD309^+^Tie^2+^, were identified as a multivariate one-step backward logistic regression analysis. There was a significant effect of cardiovascular risk factors (T2DM, SUA, TC, hs-CRP, LDL-cholesterol) and Agatston score index on the combined dependent variable (CD45^−^CD34^+^, CD14^+^CD309^+^, and CD14^+^CD309^+^Tie^2+^ cell subsets) (*F* = 46.16, *p* < 0.001; Wilks’ Lambda = 0.05; partial η^2^ = 0.72). Analysis of each individual dependent variable showed that there was an effect of hs-CRP level (*F* = 0.39, *p* = 0.002, partial η^2^ = 0.52), T2DM (*F* = 0.38, *p* = 0.016, partial η^2^ = 0.33), LDL-cholesterol (*F* = 0.38, *p* = 0.018, partial η^2^ = 0.30) on CD14^+^CD309^+^ cells. A significant effect of T2DM (*F* = 0.41, *p* = 0.001, partial η^2^ = 0.60), SUA (*F* = 0.32, *p* = 0.024, partial η^2^ = 0.32), hs-CRP (*F* = 0.36, *p* = 0.008, partial η^2^ = 0.70), LDL-cholesterol (*F* = 0.36, *p* = 0.004, partial η^2^ = 0.32), and Agatston score index (*F* = 0.34, *p* = 0.004, partial η^2^ = 0.31) on CMCs determined as CD14^+^CD309^+^Tie^2+^ was found. There was a relation between cardiovascular risk factors and CMCs determined as CD45^−^CD34^+^, while an effect of Agatston score index to CD45^−^CD34^+^ cell subset (*F* = 0.34, *p* = 0.028, partial η^2^ = 0.36) was supposed. 

**Table 3:** Frequencies and absolute values of circulating mononuclear cells in the study patient population.

	Healthy volunteers (*n* = 25)	Patients with known CAD (*n* = 126)	*p* Value
CD45^+^CD34^+^, %	1.90 (IQR = 1.49–2.10)	2.19 (IQR = 1.76–2.613)	0.36
CD45^+^CD34^+^, cells × 10^3^/μL	0.114 (IQR = 0.095–0.120)	0.113 (IQR = 0.094–0.119)	0.72
CD45^−^CD34^+^ × 10^−4^, %	1.00 (IQR = 0.69–1.35)	1.09 (IQR = 1.00–1.348)	0.15
CD45^−^CD34^+^, cells × 10^−1^/μL	0.06 (IQR = 0.05–0.07)	0.057 (IQR = 0.053–0.065)	0.12
CD14^+^CD309^+^ × 10^−4^, %	71.00 (IQR = 61.50–96.00)	57.00 (IQR = 43.20–81.50)	0.02
CD14^+^CD309^+^, cells × 10^−1^/μL	4.26 (IQR = 3.70–5.74)	2.96 (IQR = 2.25–4.21)	0.01
CD14^+^CD309^+^Tie^2+^ × 10^−4^, %	7.70 (IQR = 4.20–12.20)	5.50 (IQR = 3.05–8.15)	0.04
CD14^+^CD309^+^Tie^2+^, cells × 10^−1^/μL	0.465 (IQR = 0.253–0.710)	0.270 (IQR = 0.241–0.411)	0.01

Note: The values corespond to medians and a interquartile range (IQR) of 25%–75%. Statistical comparisons are made using Mann-Whitney test with significance levels of 0.05 and 0.01 (for 2-tailed). Note: Values are medians and 25%–75% interquartile range (IQR). Statistical comparisons are made.

The odds ratio (OR) and confidence intervals (95% CI) were calculated for factors independently associated with CMC lowering (Table 4).

**Table 4:** Independent predictors of circulating circulating mononuclear cells (CMCs). The results of multivariate one-step backward logistic regression analysis.

Factors	CD14^+^CD309^+^		CD14^+^CD309^+^Tie^2+^	
	OR (95% CI)	*p* Value	OR (95% CI)	*p* Value
Gender (male)	0.82 (0.71–1.02)	0.043	1.03 (1.00–1.10)	0.042
Hypertension	0.93 (0.80–1.03)	0.025	1.05 (0.97–1.11)	0.005
Hyperlipidemia	1.04 (0.92–1.22)	0.032	1.09 (1.04–1.18)	0.034
T2DM	1.18 (1.10–1.31)	0.005	1.20 (1.06–1.34)	0.005
BMI	0.95 (0.74–1.18)	0.039	1.03 (0.99–1.07)	0.052
Smoking	0.80 (0.55–1.16)	0.042	0.92 (0.72–1.01)	0.006
hs-CRP > 2.54 mg/L	1.12 (1.03–1.20)	0.007	1.22 (1.06–1.44)	0.006
Agatston score index	1.14 (1.02–1.18)	0.009	1.16 (1.10–1.22)	0.044
TC	1.03 (0.88–1.13)	0.022	1.06 (1.00–1.11)	0.006
SUA	1.02 (0.94–1.14)	0.039	1.10 (0.96–1.29)	0.032
Number of MCRFs >3	1.25 (1.07–1.46)	0.008	1.27 (1.10–1.42)	0.009

Note: OR—odds ratio, CI—confidence interval, MCRFs—multiple cardiovascular risk factors.

Since no significant correlations between CD45^−^CD34^+^ and cardiovascular risk factors were found in patients with asymptomatic known CAD, multivariate statistics with OR calculations for CMCs determined as CD14^+^CD309^+^ and CD14^+^CD309^+^Tie^2+^ was performed. Analysis of the data obtained revealed that subjects with T2DM and calcification of coronary arteries determined with Agatston score index had a 1.16-fold and 1.14-fold increase in OR of CD14^+^CD309^+^ CMC lowering in circulation respectively. When three and more cardiovascular risk factors occurred, circulating CD14^+^CD309^+^ CMCs lowered more significantly (OR = 1.27; 95% CI = 1.10–1.42; *p* = 0.009). Contrary to expectations, no significant independent correlation between CMC concentration, and the number of HD-NCP and LD-NCP in the coronary arteries was found. There were a number of cardiovascular risk factors that had an effect on CD14^+^CD309^+^Tie^2+^ CMC lowering. The most important factors were T2DM (OR = 1.20; 95% CI = 1.06–1.34; *p* = 0.005), hs-CRP >2.54 mg/L (OR = 1.22; 95% CI = 1.06–1.44; *p* = 0.006), Agatston score index (OR = 1.16; 95% CI = 1.10–1.22; *p* = 0.044) and the number of MCRF >3 (OR = 1.27; 95% CI = 1.10–1.42; *p* = 0.009). Finally, the conclusion was made that it is CD14^+^CD309^+^Tie^2+^ CMCs lowering that demonstrated a more pronounced fit with MCRF in cohort of the asymptomatic patients with known CAD.

Recent studies show that bone marrow-derived endothelial progenitor cells (EPCs) play an important role in the maintenance of endothelial integrity and atherosclerosis [3,14]. A correlation between MCRF and concentration of CD34^+^CD45^−^ cells and CD14^+^CD309^+^/CD14^+^CD309^+^Tie^2+^ cells was found in asymptomatic coronary artery disease patients. Recently a clinical study showed that there is inverse correlation between the number of circulating CMCs and cardiovascular risk factors [24]. nevertheless, as the number of the existing cardiovascular risk factors varies among patients, simple CMC counts do not describe adequately a vascular disease risk in any clinical condition and, therefore, the CVD risk remains [24,25]. The authors agree with Padfield G. J. *et al*. [15] who showed that CD34^+^CD45^−^ cell number was increased in patients with CAD compared with those with normal coronary arteries and correlated well with atheroma. The authors believed that CD14^+^VEGFR-2^+^Tie-2^+^ cells and endothelial cell-colony forming units were increased in patients with acute coronary syndrome; that cell concentrations reflected myocardial necrosis, and did not predict the extent of CAD. Patients with myocardial infarction or acute coronary syndrome, however, were not included in this study. The authors also determined that there was a reduction in CD14^+^VEGFR-2^+^Tie-2^+^ concentration, as well as a trend of an increase in CD34^+^CD45^−^ cells in the cohort of asymptomatic patients with coronary calcification and known CAD at the early stage. The authors agreed that no exact fit exists between the concentration of the EPCs traditionally determinined and severity of coronary atherosclerosis. The data obtained reflect that the concentration of monocytes with proangiogenic capacity, which were determined as CD14^+^CD309^+^/CD14^+^CD309^+^Tie^2+^ cell subsets, is associated more with the number of cardiovascular risk factors than with the calcification of the coronary arteries and plaques types. MCRFs, however, were powerful predictors of circulating CD14^+^CD309^+^/CD14^+^CD309^+^Tie^2+^ cells irrespective of the severity of coronary calcification. Provided by Chen, J. Z. *et al*. [7] and Loomans, C. J. *et al*. [2], the results clarified a pivotal role of CMC lowering in pathogenesis of vascular complication in patients with diabetes and hyperlipidaemia. Werner, N. and Nickenig, G. [26] demonstrated a correlation between lower CD34^+^/CD309^+^ cells in later stages of CAD. Boilson, B. A., Kiernan, T. J., Harbuzariu, A., Nelson, R. E., Lerman, A. and Simari, R. D. [27], presented data which supported this correlation available at a much earlier stage of CAD. Obtained in this study, the data show that MCRFs had the most important prediction value to lower circulating CD14^+^CD309^+^/CD14^+^CD309^+^Tie^2+^ cells when compared with isolated cardiovascular risk factors, even those such as T2DM and hs-CRP elevation in asymptomatic patients with known CAD. These findings may support a hypothesis of the impact of MCRFs on disorders of cellular protective mechanisms in subjects at an early stage of CAD.

## 5. Conclusion

The authors postulate that a reduction in circulating CD14^+^CD309^+^ and CD14^+^CD309^+^Tei^2+^ EPCs is related to a number of cardiovascular risk factors in asymptomatic patients with known CAD.

## 6. Study Restrictions

This study has some restrictions. The authors believe that a greater cohort is desirable to improve the power of the study. There is a variation in the definition of EPCs, the number of existing cardiovascular risk factors in various patients, and in the interaction between EPCs and other hematopoietic progenitor, inflammatory cells or platelets. The authors suppose that these restrictions might have no significant impact on the study data interpretation.

## References

[1] Krankel N., Adams V., Linke A., Gielen S., Erbs S., Lenk K., Schuler G., Hambrecht R. (2005). Hyperglycemia reduces survival and impairs function of circulating blood-derived progenitor cells. Arterioscler. Thromb. Vasc. Biol..

[2] Loomans C.J., de Koning E.J., Staal F.J., Rookmaaker M.B., Verseyden C., de Boer H.C., Verhaar M.C., Braam B., Rabelink T.J., van Zonneveld A.J. (2004). Endothelial progenitor cell dysfunction: A novel concept in the pathogenesis of vascular complications of type 1 diabetes. Diabetes.

[3] Singh N., van Craeyveld E., Tjwa M., Ciarka A., Emmerechts J., Droogne W., Gordts S.C., Carlier V., Jacobs F., Fieuws S. (2012). Circulating apoptotic endothelial cells and apoptotic endothelial microparticles independently predict the presence of cardiac allograft vasculopathy. J. Am. Coll. Cardiol..

[4] Sobrino T., Hurtado O., Moro M.A., Rodríguez-Yáñez M., Castellanos M., Brea D., Moldes O., Blanco M., Arenillas J.F., Leira R. (2007). The increase of circulating endothelial progenitor cells after acute ischemic stroke is associated with good outcome. Stroke.

[5] Ravi S., Caves J.M., Martinez A.W., Xiao J., Wen J., Haller C.A., Davis M.E., Chaikof E.L. (2012). Effect of bone marrow-derived extracellular matrix on cardiac function after ischemic injury. Biomaterials.

[6] George J., Goldstein E., Abashidze S., Deutsch V., Schmilovich H., Finkelstein A., Herz I., Miller H., Keren G. (2004). Circulating endothelial progenitor cells in patients with unstable angina: Association with systemic inflammation. Eur. Heart J..

[7] Chen J.Z., Zhang F.R., Tao Q.M., Wang X.X., Zhu J.H., Zhu J.H. (2004). Number and activity of endothelial progenitor cells from peripheral blood in patients with hypercholesterolaemia. Clin. Sci. (Lond.).

[8] Adams V., Lenk K., Linke A., Lenz D., Erbs S., Sandri M., Tarnok A., Gielen S., Emmrich F., Schuler G. (2004). Increase of circulating endothelial progenitor cells in patients with coronary artery disease after exercise-induced ischemia. Arterioscler. Thromb. Vasc. Biol..

[9] Banerjee S., Brilakis E., Zhang S., Roesle M., Lindsey J., Philips B., Blewett C.G., Terada L.S. (2006). Endothelial progenitor cell mobilization after percutaneous coronary intervention. Atherosclerosis.

[10] Hill J.M., Zalos G., Halcox J.P., Schenke W.H., Waclawiw M.A., Quyyumi A.A., Finkel T. (2003). Circulating endotelial progenitor cells, vascular function, and cardiovascular risk. N. Engl. J. Med..

[11] George J., Shmilovich H., Deutsch V., Miller H., Keren G., Roth A. (2006). Comparative analysis of methods for assessment of circulating endothelial progenitor cells. Tissue Eng..

[12] Vasa M., Fichtlscherer S., Aicher A., Adler K., Urbich C., Martin H., Zeiher A.M., Dimmeler S. (2001). Number and migratory activity of circulating endothelial progenitor cells inversely correlate with risk factors for coronary artery disease. Circ. Res..

[13] Tamura H., Okamoto S., Iwatsuki K., Futamata Y., Tanaka K., Nakayama Y., Miyajima A., Hara T. (2002). *In vivo* differentiation of stem cells in the aorta-gonad-mesonephros region of mouse embryo and adult bone marrow. Exp. Hematol..

[14] Morishita T., Uzui H., Nakano A., Mitsuke Y., Geshi T., Ueda T., Lee J.D. (2012). Number of endothelial progenitor cells in peripheral artery disease as a marker of severity and association with pentraxin-3, malondialdehyde-modified low-density lipoprotein and membrane type-1 matrix metalloproteinase. J. Atheroscler. Thromb..

[15] Padfield G.J., Tura-Ceide O., Freyer E., Barclay G.R., Turner M., Newby D.E., Mills N.L. (2013). Endothelial progenitor cells, atheroma burden and clinical outcome in patients with coronary artery disease. Heart.

[16] Bluemke D.A., Achenbach S., Budoff M., Gerber T.C., Gersh B., Hillis L.D., Hundley W.G., Manning W.J., Printz B.F., Stuber M. (2008). Noninvasive coronary artery imaging: Magnetic resonance angiography and multidetector computed tomography angiography: A scientific statement from the American heart association committee on cardiovascular imaging and intervention of the council on cardiovascular radiology and intervention, and the councils on clinical cardiology and cardiovascular disease in the young. Circulation.

[17] Agatston A.S., Janowitz W.R. (1994). Ultrafast computed tomography in coronary screening. Circulation.

[18] Budoff M.J., Achenbach S., Blumenthal R.S., Carr J.J., Goldin J.G., Greenland P., Guerci A.D., Lima J.A.C., Rader D.J., Rubin G.D. (2006). Assessment of coronary artery disease by cardiac computed tomography: A scientific statement from the American Heart Association Committee on Cardiovascular Imaging and Intervention, Council on Cardiovascular Radiology and Intervention, and Committee on Cardiac Imaging, Council on Clinical Cardiology. Circulation.

[19] Agatston A.S., Janowitz W.R., Hildner F.J., Zusmer N.R., Viamonte M., Detrano R. (1994). Quantification of coronary artery calcium using ultrafast computed tomography. J. Am. Coll. Cardiol..

[20] Schiller N.B., Shah P.M., Crawford M., de Maria A., Devereux R., Feigenbaum H., Gutgesell H., Reichek N., Sahn D., Schnittger I. (1989). Recommendations for quantitation of the left ventricle by two-dimensional echocardiography. American Society of Echocardiography Committee on Standards, Subcommittee on Quantitation of Two-Dimensional Echocardiograms. J. Am. Soc. Echocardiogr..

[21] Levey A.S., Stevens L.A., Schmid C.H., Zhang Y.L., Castro A.F., Feldman H.I.,  Kusek J.W., Eggers P., van Lente F., Greene T. (2009). A new equation to estimate glomerular filtration rate. Ann. Intern. Med..

[22] Friedewald W.T., Levy R.I., Fredrickson D.S. (1972). Estimation of the concentration of low-density lipoprotein cholesterol in plasma, without use of the preparative ultracentrifuge. Clin. Chem..

[23] Tung J.W., Parks D.R., Moore W.A., Herzenberg L.A., Herzenberg L.A. (2004). New approaches to fluorescence compensation and visualization of FACS data. Clin. Immunol..

[24] Bakogiannis C., Tousoulis D., Androulakis E., Briasoulis A., Papageorgiou N., Vogiatzi G., Kampoli A.M., Charakida M., Siasos G., Latsios G. (2012). Circulating endothelial progenitor cells as biomarkers for prediction of cardiovascular outcomes. Curr. Med. Chem..

[25] Liew A., Barry F., O’Brien T. (2006). Endothelial progenitor cells: Diagnostic and therapeutic considerations. Bioessays.

[26] Werner N., Nickenig G. (2006). Influence of cardiovascular risk factors on endothelial progenitor cells: Limitations for therapy?. Arterioscler. Thromb. Vasc. Biol..

[27] Boilson B.A., Kiernan T.J., Harbuzariu A., Nelson R.E., Lerman A., Simari R.D. (2008). Circulating CD34^+^ cell subsets in patients with coronary endothelial dysfunction. Nat. Clin. Pract. Cardiovasc. Med..

